# A novel kinesin involved in flagellum attachment and positioning in *Trypanosoma brucei*

**DOI:** 10.1186/2046-2530-4-S1-P44

**Published:** 2015-07-13

**Authors:** S Luiggi, A Raïa, L Petit, T Blisnick, S Perrot, L Pao, P Bastin, P Grellier, L Kohl

**Affiliations:** 1 Biodiversity and Adaptation of Eukaryotic Microorganisms to their Environment, UMR7245 MNHN/CNRS, National Museum of Natural History, Paris, France; 2 Trypanosome Cell Biology Unit, Pasteur Institute, Paris, France

## Objective

Kinesins are motor proteins that transport cargo along microtubules using ATP and fulfil important roles in cilia and flagella. KIN5 is an orphan kinesin, i.e. it does not belong to any of the known kinesin families, and it is found only in trypanosomatids.

## Methods

The function of this protein has been investigated by inducible RNA interference (RNAi) followed by phenotypic characterisation, in the protist *Trypanosoma brucei*, which possesses a single attached flagellum. Its localisation was determined by expressing a hybrid YFP::KIN5 protein.

## Results

In procyclic cells KIN5 is localised in the flagellum (probably the axoneme), with a strong fluorescent signal at the distal tip. Depletion of KIN5 results in cells with a mispositioned and partially detached flagellum. The flagellum of the cells is still beating, but the cells are unable to swim. Nevertheless they divide normally indicating that they have adapted to the partially detached flagellum, probably by modifications of the intracellular organisation. Preliminary data indicate that the filament of the Flagellum Attachment Zone is smaller in length in cells depleted of KIN5.

## Conclusion

KIN5 is a flagellar kinesin involved flagellum positioning/attachment, which is not essential for cell survival in procyclic cells in culture. We hypothesise that the cells have adapted their intracellular organisation to allow replication and cell division. We now investigate the relationship between KIN5 and other proteins known to be involved in flagellum attachment.

